# Dexmedetomidine inhibits inflammation in microglia cells under stimulation of LPS and ATP by c-Fos/NLRP3/caspase-1 cascades

**DOI:** 10.17179/excli2017-1018

**Published:** 2018-03-22

**Authors:** Hu Li, Xueping Zhang, Mingfu Chen, Jianyan Chen, Tao Gao, Shanglong Yao

**Affiliations:** 1 Department of Anesthesiology, Shenzhen Baoan Hospital Affiliated to Southern Medical University, Shenzhen 518100, China; 2 Department of Anesthesiology, Shenzhen People's Hospital, The Second Clinical Medical College, Jinan University, Shenzhen Anesthesiology Engineering Center, Shenzhen 518020, China; 3 Department of Anesthesiology, Shenzhen Shajin Hospital Affiliated to Guangzhou Medical University, Shenzhen 518100, China; 4 Department of Anesthesiology, Second People's Hospital of Futian District, Shenzhen 518029, China; 5 Department of Anesthesiology, Union Hospital, Tongji Medical College, Huazhong University of Science and Technology, Wuhan 430022, China

**Keywords:** dexmedetomidine, NLRP3, inflammatory, microglia

## Abstract

NOD-like receptor 3 (NLRP3) plays critical roles in the initiation of inflammasome-mediated inflammation in microglia, thus becomes an important therapeutic target of Alzheimer's disease (AD). Dexmedetomidine (Dex), a new type of clinical anesthetic agent, shows anti-inflammatory properties and inhibits postoperative cognitive dysfunction in AD patients. The present study was aimed to investigate effect of Dex on NLRP3 activity in activated microglia and reveal the underlying mechanisms. The human microglia clone 3 (HMC3) cells were exposed to 100 ng/ml LPS and 5 mM ATP, in the presence and absence of doses of Dex. Data from ELISA and Western blot assays showed that Dex abrogated the promoting effects of LPS/ATP on the release of pro-inflammatory cytokines including IL-1β and IL-18 in the cell medium and the expression of NLRP3 and its downstream target caspase-1 in HMC3 cells. Furthermore, the present study found that exposure of HMC3 cells to LPS/ATP increased nuclear protein levels of transcription factor c-Fos, but treatment with Dex reversed the increase in c-Fos, as indicated by Western blot and immunofluorescence measures. Luciferase reported assay revealed that c-Fos can bind to the promoter region of *NLRP3* gene and positively regulate the expression. These results suggest that Dex inhibiting c-Fos nuclear protein levels promoted by LPS/ATP blocks the up-regulation of NLRP3. This suggestion is supported by co-immunoprecipitation and PCR studies, in which Dex decreased the amount of c-Fos that binds to NLRP3 under the stimulation of LPS/ATP. The present study revealed that Dex inhibits inflammation in microglia cells under stimulation of LPS and ATP by c-Fos/NLRP3/caspase-1 cascades, which adds new understanding of the anti-inflammatory mechanism of Dex.

## Introduction

Alzheimer's disease (AD) is a prevalent neurodegenerative disease, which involves progressive memory loss, cognitive impairment, behavioral deficits and dementia (Vallée and Lecarpentier, 2016[20]). Microglial cells are the resident immuno-competent cells of the central nervous system (CNS), responsible for sensing any perturbation in the CNS and orchestrating the immune response (Udeochu et al., 2016[19]). In addition, they actively participate in CNS wiring and modulation of neuronal activities (Schafer et al., 2012[16]). However, uncontrolled or chronic activation of microglia is closely correlated to the pathogenesis of AD (Ramirez et al., 2017[14]). A local activation of microglial cells is consistently detected in the brains of AD patients (Ramirez et al., 2017[14]). The release of pro-inflammatory mediators from activated microglia is potentially harmful and can cause cellular damage. There is evidence indicating that microglial activation is associated with deposition of amyloid-beta (Aβ), formation of amyloid plaques (senile plaques) and neurofibrillary tangles (Ramirez et al., 2017[14]). 

NOD-like receptors (NLRs), a class of cytosolic sensors or receptors, play critical roles in the initiation of inflammasome-mediated neuroinflammation in microglia (Freeman and Ting, 2016[3]). NLRs can detect and response to pathogen-associated molecular patterns (PAMPs), which are linked to bacterial, viral and fungal, as well as damage-associated molecular patterns (DAMPs) that are produced during tissue and cell damages (Freeman and Ting, 2016[3]). NLRP3 is one of the most extensively studied classes of NLRs. Activated NLRP3 triggers the cleavage of pro-caspase-1 into its active and mature form caspase-1. Caspase-1 then induces a secondary cascade of events associated to releases of pro-inflammatory cytokines such as IL-1β and IL-18 cytokines (Saresella et al., 2016[15]). These cytokines are known to cause the proliferation and activation of neuroinflammatory cells such as microglia and astrocytes. As NLRP3 represents a critical mediator of neuroinflammation, it becomes an important therapeutic target of AD and other neurodegenerative diseases (Saresella et al., 2016[15]). 

Converging evidence suggests that administration of general anesthesia (e.g. isoflurane-mediated anesthesia) increases the risk of AD. An increased incidence of AD in older patients undergoing general anesthesia has been found by scholars studying the relationship between anesthesia and AD (Xie et al., 2006[21]; Planel et al., 2009[13]). In addition, isoflurane anesthesia is associated to increased postoperative cognitive dysfunction (POCD) in AD patients and aged people (Zhang et al., 2017[26]). Dexmedetomidine (Dex), a specific agonist of α2-adrenoceptor, has been used as a new type of clinical anesthetic agent especially for calmness of tracheal intubation and mechanical ventilation in surgical patients under general anesthesia (Liu et al., 2017[10]). Dex is one of the few anesthetics that show inhibitory effect on POCD and neuroprotective actions against brain ischemia (Zhou et al., 2016[27]; Liu et al., 2017[10]). Furthermore, an emerging literature shows that Dex inhibits NLRP3 inflammasome activation, and consequently alleviates hyperoxia-induced acute lung injury in an *in vivo* model, which suggests that Dex also has anti-inflammatory properties (Zhang et al., 2017[25]). The present study investigated whether Dex has the capacity to inhibit NLRP3 activity in activated microglia to reveal the potential neuroprotective effect against AD. 

## Materials and Methods

### Cell culture and treatments

The human microglia clone 3 cell line (HMC3) was purchased from American Type Culture Collection (ATCC, Rockville, MD, USA). HMC3 cells were initially produced by transfecting human embryonic brain-derived macrophages with the large T antigen of the simian virus 40. HMC3 cells were cultured in Eagle's Minimum Essential Media (Thermo Fisher Scientific, Shanghai, China) supplemented with 10 % fetal calf serum (Sigma-Aldrich, St. Louis, MO, USA) and 100 units/ml (U/ml) penicillin/streptomycin (Invitrogen, Carlsbad, CA, USA), and incubated in a humidified atmosphere (5 % CO_2_) at 37 °C. Cells were plated in 24-well plates at 2 × 10^5^ cells/well at least for 24 h before treatment with pharmacological substances. 

HMC3 cells were activated by culturing in the medium with lipopolysaccharide (LPS, 100 ng/ml, Sigma-Aldrich) for 24 h and with adenosine triphosphate (ATP, 5 mM, Sigma-Aldrich) for additional 30 min. To determine the anti-inflammatory effects of Dex (Sigma-Aldrich) in HMC3 cells, these cells were treated with various concentrations of Dex (0, 0.1, 1, or 10 µM) when exposing to LPS/ATP. ELISA assay was performed to evaluate concentrations of pro-inflammatory cytokines, including IL-1β and IL-18, in the media.

### Cell viability

Cell viability was analyzed by 3-(4,5-dimethylthiazol-2-yl)-2,5-diphenyltetrazolium bromide (MTT) assay. HMC3 cells were seeded into each well of a 96-well micro-plate. After treatments, MTT (Beyotime Institute of Biotechnology, Nanjing, China) solution was added to the cells with a final concentration of 1 mg/mL and the mixture was allowed to incubate at 37 °C for about 4 h. The supernatant was removed, and then the pellets were dissolved in dimethyl sulfoxide (Beyotime Institute of Biotechnology). The absorbance was measured by spectrophotometry at 570 nm using an Elx-800uv reader (Bio-Tek Instruments, Winooski, VT, USA). 

### ELISA assay

Culture supernatants were collected and stored at −20 °C until analysis. The concentrations of IL-1β and IL-18 cytokines in cell culture supernatants were measured using ELISA kits (R&D Systems, Minneapolis, MN, USA) according to the manufacturer's instructions.

### Western blot analysis

Cellular whole protein and nuclear protein were extracted respectively using the Protein Extraction Kit (Sigma, St Louis, MO, USA) and NE-PER Nuclear Extraction Reagents (Thermo Scientific, Rockford, IL, USA) according to the manufacturers' protocols. The protein concentration in the supernatant fluid of the lysate was measured by the BCA protein assay (Pierce, Rockfold, IL, USA). Equal amounts of protein (20 μg) in each well were loaded for electrophoresis in 12 % sodium dodecyl sulfate-polyacrylamide gels, and then gels were transferred to polyvinylidene fluoride microporous membranes (PVDF; Millipore, Bedford, MA, USA). Membranes containing the transferred proteins were blocked with 5 % skim milk in TBS containing 0.1 % Tween-20 (TBST) for 2 hours at room temperature. The membranes were hybridized with antibodies against NLRP3 (Dilution 1:500, ab214185, Abcam, Shanghai, China), caspase-1 (Dilution 1:800, ab14367, Abcam), c-Fos (Dilution 1:500, ab134122, Abcam), Histone (Dilution 1:1000, sc-56616, Santa Cruz Biotechnology, Santa Cruz, CA, USA) and GAPDH (Dilution 1:1000, sc-365062, Santa Cruz Biotechnology) at 4 °C overnight. The primary antibodies were visualized by adding secondary biotin-conjugated antibodies followed by an avidin/biotin/peroxidase complex (Vectastain ABC Elite kit; Vector Laboratories Inc, Burlingame, CA, USA) and substrate (Vector Nova RED, Vectastain).

### c-Fos overexpression and knockdown 

An open-reading frame clone of* Homo sapiens c-fos *gene was inserted into the enhanced green fluorescent protein plasmid-C1 (pEGFP-C1) vector (GenePharma Co., Ltd) to construct over-expression vectors of c-Fos. The c-Fos vectors were transfected into HMC3 cells using Lipofectamine TM 2000 (Invitrogen, Carlsbad, CA, USA), according to the manufacturer's instructions. HMC3 cells that were transfected with the empty vectors were used as the control. Small interfering RNA (siRNA) oligonucleotides against c-Fos (siRNA-c-Fos) and the corresponding scrambled control oligonucleotides were synthesized from Genephama Biotech (Shanghai, China). For c-Fos knockdown, 60 nM siRNA-c-Fos was transfected into HMC3 cells using X-tremeGENE siRNA Transfection Reagent (Roche, Germany) according to the manufacturer's protocol.

### Luciferase reporter assay

The promoter region of *NLRP3* gene was cloned and inserted into pGL3-basic vector (Promega, Madison, Wisconsin, USA) at site between XhoI and SacI to form pGL3-NLRP3-promter construct. Within 48 h of passage and more than 80% confluent, HMC3 cells were transfected with pGL3-NLRP3-promter constructs, pGL3-NLRP3-promter constructs + c-Fos vectors, pGL3-basic vectors, or pGL3-promoter constructs using Lipofectamine TM 2000 (Invitrogen) in 24-well plates. Each well included 1.5×10^5^ cells, 1 mg indicated constructs, 0.04 mg internal control vector pRL-TK, 2 ml Lipofectamine TM 2000 and 500 ml culture media without serum and antibiotics. All cells were analyzed for dual-luciferase reporter activity 48 h after completion of the transfection procedure, using a Sirius luminometer (Berthold Detection System GmbH, Pforzheim, Germany). 

### Immunofluorescence (IF) assay 

The HMC3 cells were fixed with 4 % paraformaldehyde for 15 min and blocked with PBS containing 0.3 % Triton X-100/5 % BSA (w/v) for 1 h at room temperature. The cells were immunostained with c-Fos antibody (Dilution 1:800, Abcam) at 37 °C for 2 h, and then incubated with secondary antibody (1:1000) and DAPI (0.3 μM). Fluorescent intensity was evaluated using a Zeiss Axioskop2 Microscope and Axiovision v4.8.1 software (Carl Zeiss MicroImaging, Inc. Weimar, Germany). 

### Co-immunoprecipitation (Co-IP) and PCR assays

Nucleoprotein in HMC3 cells was extracted with a CelLytic™ NuCLEAR™ Extraction Kit (Sigma-Aldrich; Merck KGaA, Germany) and incubated c-Fos primary antibody (1:500 dilution; Santa Cruz Biotechnology, Inc.) at 4 °C for 60 min with gentle mixing. Subsequently, 20 µl Protein A/G Plus-Agarose beads (Thermo Fisher Scientific, Inc.) were added, followed by incubation at 4 °C overnight. The mixture was centrifuged at 500 x g for 5 min at 4 °C. The supernatant was discarded and the Co-IP products were washed three times with PBS. After the final wash, the precipitates were re-suspended in 40 μl sample buffer and detected by PCR to quantify the NLRP3 gene expression. 

PCR was performed using the iQ5 Multicolor Real-Time PCR Detection System (Bio-Rad Laboratories Inc., Hercules, CA, USA) with SYBR Premix Ex Taq II (Takara). The PCR primer sequences for NLRP3 and GAPDH were as follows: NLRP3, (F)5'-GCTGGTCTTGAATTCCTCA-3' and (R) 5'-GGCACACGGATGAGTCTTT-3'; GAPDH, (F) 5'-TACTAGCGGTTTTACGGGCG-3' and (R) 5'- TCGAACAGGAGGAGCAGAGAGCGA-3'. A melting curve analysis of the amplified products was performed at the end of each PCR cycle. The comparative C(T) method was used to quantify the expression of NLRP3 using GAPDH as the normalization control.

### Statistical analysis

Data were expressed as mean ± SD (range) for the parametric variables, analyzed using SAS9.0 software (SAS Institute, USA). Analysis of variance (ANOVA) was performed to compare the difference between multiple groups using t test. *P* value <0.05 was regarded as significant.

## Results

### Dex inhibits inflammatory responses of HMC3 cells induced by LPS/ATP

**Figure 1:**
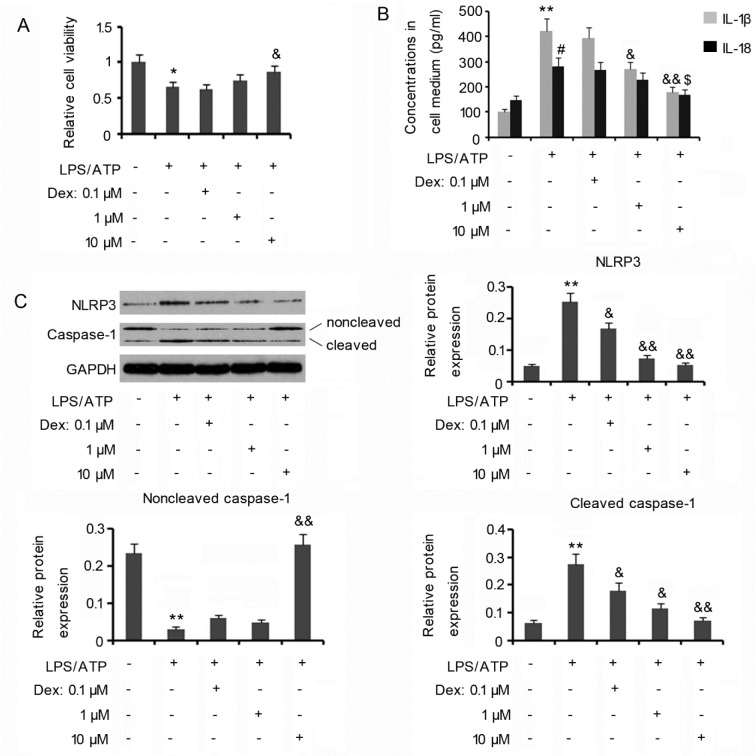
Dex inhibits inflammatory responses of HMC3 cells induced by LPS/ATP HMC3 cells were exposed to 100 ng/ml LPS and 5 mM ATP, in the presence and absence of doses of Dex. The cells were then subjected to the measurement of cell viability (A). ELISA (B) and Western blot assays (C) assessed the concentrations of pro-inflammatory cytokines including IL-1β and IL-18 in the cell medium and the expression of NLRP3 and its downstream target caspase-1 in the cells, respectively. *P<0.05, **P<0.01, ^#^*P*<0.05 and ^##^*P*<0.01*vs.* control; ^&^*P*<0.05, ^&&^*P*<0.01, ^$^*P*<0.05 and ^$$^*P*<0.01*vs.* LPS/ATP group. Dex: Dexmedetomidine

In the present study, microglia HMC3 cells were activated by culturing in the medium containing LPS and ATP. Activated HMC3 cells showed decreased cell viability (p<0.05, Figure 1A(Fig. 1)) and increased release of inflammatory factors, including IL-1β (p<0.01, Figure 1B(Fig. 1)) and IL-18 (p<0.05), compared with non-activated cells. To understand the anti-inflammatory effect of Dex in HMC3 cells, these cells were treated with various concentrations of Dex (0, 0.1, 1, or 10 µM) when exposing to LPS/ATP. The inhibited cell viability was notably restored by 10 µM Dex (p<0.05 vs. LPS/ATP group, Figure 1A(Fig. 1)), but not by 0.1 and 1 µM Dex. Adding 1 (p<0.05 vs. LPS/ATP group, Figure 1B(Fig. 1)) and 10 µM Dex (p<0.01 vs. LPS/ATP group) to cells decreased the increase of IL-1β release by LPS/ATP. The increase in IL-18 release by LPS/ATP was also decreased by 10 µM Dex (p<0.05 vs. LPS/ATP group). 

NLRP3/caspase-1 cascades mediate the production and release of inflammatory factors, such as IL-1β and IL-18, following the stimulation of LPS/ATP (Schafer et al., 2012[16]). Western blot assay revealed that treatment with LPS/ATP indeed increased NLRP3 expression in HMC3 cells (p<0.01 Figure 1C(Fig. 1)), however the increase in NLRP3 expression was reduced by 0.1 (p<0.05), 1 (p<0.01) and 10 µM Dex (p<0.01). Activated NLRP3 triggers the cleavage of pro-caspase-1 into its active and mature form caspase-1 (Schafer et al., 2012[16]). Protein level of non-cleaved caspase-1 in HMC3 cells was decreased by treatment with LPS/ATP (p<0.01), but cleaved caspase-1 was dramatically increased (p<0.01 Figure 1C(Fig. 1)). The decreased protein level of non-cleaved caspase-1 was restored by 10 µM Dex (p<0.01 vs. LPS/ATP group, Figure 1C(Fig. 1)), but not by 0.1 and 1 µM Dex. The increase in protein level of cleaved caspase-1 was reduced by 0.1 (p<0.05), 1 (p<0.05) and 10 µM Dex (p<0.01). These data suggest that the anti-inflammatory effect of Dex is associated with the inhibition of NLRP3/caspase-1 cascades. 

### c-Fos mediates the inhibitory effect of Dex on NLRP3

**Figure 2:**
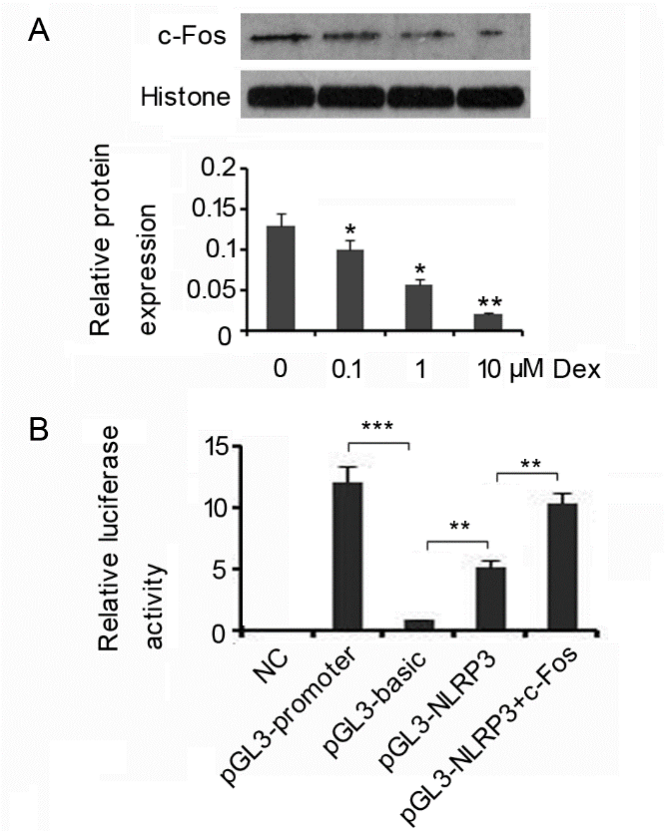
c-Fos mediates the inhibitory effect of Dex on NLRP3 HMC3 cells were treated with doses of Dex. Western blot detected nuclear protein levels of c-Fos in the HMC3 cells (A). In luciferase reporter assay, HMC3 cells were transfected with pGL3-NLRP3-promter constructs, pGL3-NLRP3-promter constructs + c-Fos vectors, pGL3-basic vectors, or pGL3-promoter constructs using Lipofectamine TM 2000 (B). *P<0.05, **P<0.01 and ***P<0.001*vs.* control. Dex: Dexmedetomidine

There is evidence indicating that various biological effects of Dex are associated with its modulating transcription factor c-Fos (Zhang et al., 2013[24]; Xiong et al., 2016[22]). The present study found that nuclear protein levels of c-Fos was dose-dependently decreased by Dex from 0.1 (p<0.05) to 10 µM (p<0.01) in HMC3 cells that were not exposed to LPS/ATP (Figure 2A(Fig. 2)). To determine that c-Fos is involved in the regulation of *NLRP3* gene expression, we performed luciferase report assay, in which a pGL3-NLRP3-promter reporter was constructed by inserting the promoter region of *NLRP3* gene into pGL3-basic vector. Results showed that the pGL3-promoter reporters, which are provided by the detection kits to be used as positive control, had notably higher luciferase activity than pGL3-basic vectors (p<0.001, Figure 2B(Fig. 2)). Similarly, the pGL3-NLRP3-promter reporter also showed higher luciferase activity than pGL3-basic vectors (p<0.01). Co-transfection with c-Fos expression vectors further increased luciferase activity of pGL3-NLRP3-promter reporters (p<0.01 vs. pGL3-NLRP3-promter). Results of luciferase report assay indicate that c-Fos serves as the transcription factor positively regulating *NLRP3* gene expression. Thus, Dex decreasing nuclear protein levels of c-Fos likely inhibits *NLRP3* gene expression. 

### Dex decreases c-Fos nuclear protein levels and subsequently inhibits NLRP3 expression under the stimulation of LPS/ATP

**Figure 3:**
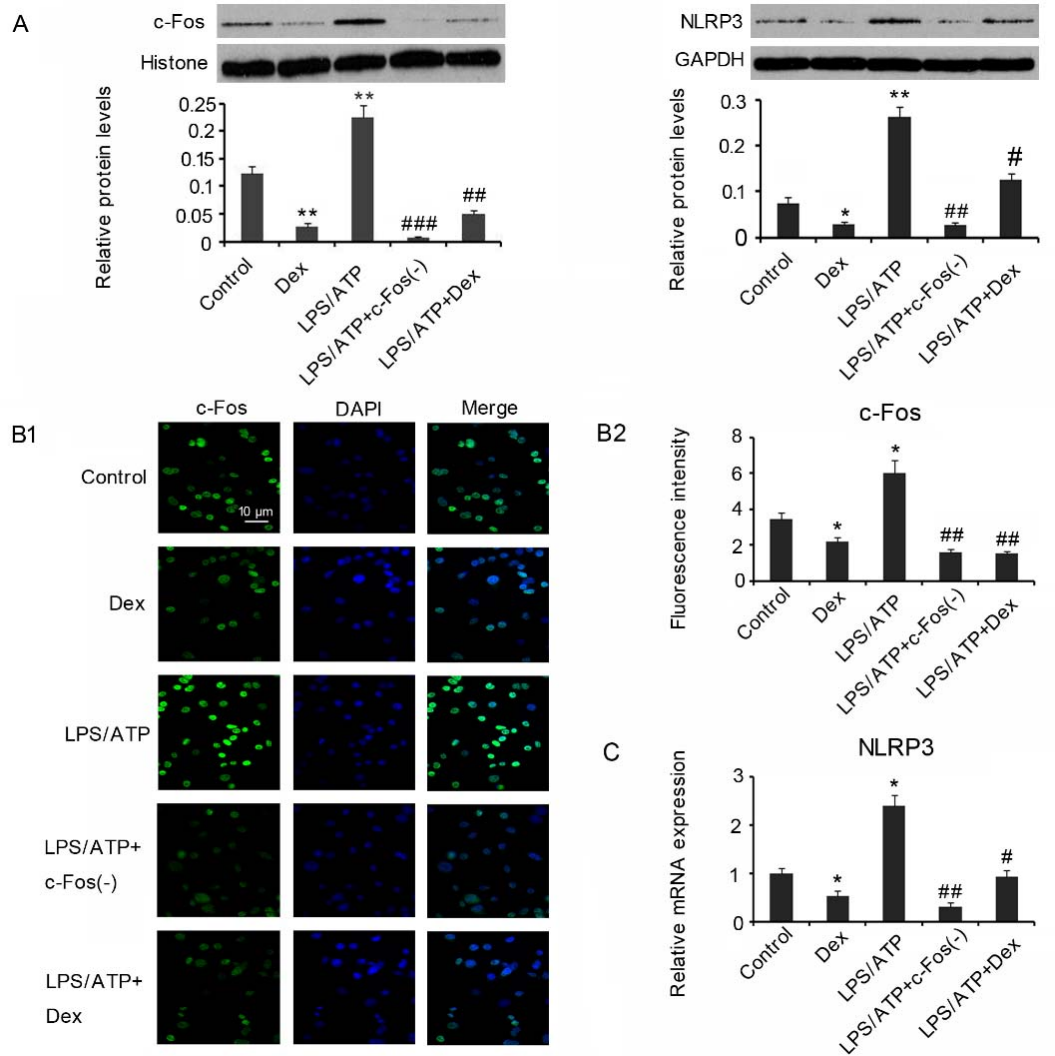
Dex inhibits c-Fos/NLRP3 cascades in microglia cells under the stimulation of LPS/ATP HMC3 cells were treated with LPS/ATP compounds and Dex alone or in combination. c-Fos was knocked down in a proportion of HMC3 cells treated with LPS/ATP compounds. Both Western blot (A) and IF assays (B) detected c-Fos nuclear protein levels and NLRP3 protein levels in the HMC3 cells. Co-IP and PCR assays (C) were performed to detect the amount of c-Fos binding to the promoter of *NLRP3* gene. *P<0.05, **P<0.01, and ***P<0.001 *vs.* control; ^#^*P*<0.05, ^##^*P*<0.01 and ^###^*P*<0.01* vs.* LPS/ATP group.

Treatment with 10 µM Dex decreased c-Fos nuclear protein levels in HMC3 cells that was not exposed to LPS/ATP (p<0.01, Figure 3A(Fig. 3)). Exposure to LPS/ATP caused remarkable increase of c-Fos nuclear protein levels in HMC3 cells. However, knockdown of c-Fos (p<0.001 vs. LPS/ATP group) or treatment with 10 µM Dex dramatically decreased c-Fos nuclear protein levels under LPS/ATP stimulation (p<0.01 vs. LPS/ATP group). NLRP3 protein level in HMC3 cells was decreased after treatment with 10 µM Dex alone (p<0.01). LPS/ATP stimulation increased NLRP3 protein levels in HMC3 cells. However, LPS/ATP induced increase in NLRP3 protein levels was reversed by c-Fos knockdown (p<0.01 vs. LPS/ATP group) and Dex treatment (p<0.01 vs. LPS/ATP group). We also performed IF assay to detect c-Fos nuclear protein levels in HMC3 cells. Dex treatment decreased c-Fos nuclear protein levels in HMC3 cells without LPS/ATP stimulation (p<0.05 vs. control, Figure 3B(Fig. 3)). LPS/ATP stimulation increased c-Fos nuclear protein levels, but the increase was abrogated by c-Fos knockdown or Dex treatment (p<0.01 vs. LPS/ATP group). Co-IP was performed to detect the amount of c-Fos binding to the promoter of *NLRP3* gene. Data from PCR assay showed decreased expression of *NLRP3* gene following Dex treatment (p<0.05, Figure 3C(Fig. 3)), which suggests decreased binding of c-Fos to the *NLRP3* gene. Treatment with LPS/ATP increased expression of *NLRP3* gene (p<0.05), suggesting increased of c-Fos to the *NLRP3* gene. c-Fos knockdown (p<0.01 vs. LPS/ATP group) and Dex treatment (p<0.05 vs. LPS/ATP group) abolished the c-Fos expression increase by LPS/ATP. 

## Discussion

The present study provided convincing evidences that Dex plays an anti-inflammatory role in human microglia cells under LPS/ATP stimulation. Chronic abnormal inflammation is correlated with amyloidosis, neurodegeneration, and memory loss in AD (Schafer et al., 2012[16]). Inflammation in AD can be induced by exogenous pathogens and sterile endogenous agents (Ishida et al., 2017[7]). LPS, an endotoxin isolated from gram-negative bacteria, can induce inflammatory responses, amyloidogenesis and neuronal damage, thus accelerating the progression of a neurodegenerative disease (Choi et al., 2017[1]). LPS has been widely used to conduct an experimental model of microglia activation (Choi et al., 2017[1]). LPS administered into brain induces microglia activation, and results in accumulation of Aβs in both the cerebral cortex and hippocampus (Sheng et al., 2013[17]). LPS increases amyloid precursor protein expression and β- and γ-secretase activities, which promotes amyloidogenesis (Sheng et al., 2013[17]). Extracellular ATP can act as a universal danger-associated molecular pattern with several known mechanisms for immune cell activation (Gilbert et al., 2016[5]; Gaikwad et al., 2017[4]). It has been demonstrated that ATP, alone or in combination with LPS, activates microglia and astrocytes and induces a neuroinflammatory response in the central nervous system (Gaikwad et al., 2017[4]; Lim et al., 2017[9]). In the present study, co-treatment with LPS and ATP stimulated the release of IL-1β and IL-18 from human microglia cells. IL-1β and IL-18 have been linked with the pathology of AD. AD patients exhibit increased levels of IL-1β and IL-18 in the serum and brain compared with the health controls (Singhal et al., 2014[18]; Demirci et al., 2017[2]). The increased generation of these proinflammatory cytokines causes microglial and astrogliosis activation and promotes the release of other pro-inflammatory molecules, resulting in unrestrained neuroinfammation (Singhal et al., 2014[18]). However, appropriate doses of Dex significantly reduced release of IL-1β and IL-18 from human microglia cells under LPS/ATP stimulation, suggesting the anti-inflammatory effect in microglia cells. 

Inflammasomes are responsible for the maturation of pro-inflammatory cytokines such as IL-1β and IL-18 (Schafer et al., 2012[16]). NLRP3 plays important roles in the initiation of inflammasome-mediated neuroinflammation in microglia (Schafer et al., 2012[16]). NLRs promote the assembly of a large multiprotein complex called the inflammasome and trigger the cleavage of pro-caspase-1 into its active and mature form caspase-1. The precursor molecules of IL-1β and IL-18 can be cleaved into their mature and active forms by caspase-1 protease (Pan et al., 2016[12]). LPS and ATP stimulation has been shown to activate NLRP3/caspase-1 cascades and consequently increases IL-1β and IL-18 production in macrophage cells (Gurung et al., 2015[6]). Inhibition of NLRP3 decreases the production of IL-1β and IL-18 both in LPS/ATP-stimulated BV2 microglia *in vitro* and spinal cord from EAE mice *in vivo* (Jiang et al., 2017[8]; Yang et al., 2018[23]). Caspase-1 inhibition decreased IL-1β production in BV2 microglial cells that are activated by LPS-treated RAW264.7 macrophages (Pan et al., 2016[12]). This evidence indicates that NLRP3/caspase-1 cascades mediates the increased production of IL-1β and IL-18 in immune cells under LPS/ATP-stimulation. Exposure to LPS/ATP also increased NLRP3 and caspase-1 expression in human microglia HMC3 cells. However, treatment with Dex abrogated the increase in NLRP3 and caspase-1 expression. Previous reports manifest that the effects of Dex against LPS-induced inflammatory response is related to the inhibition of NF-κB (Liu et al., 2016[11]). It has been acknowledged that LPS interacts with Toll-like receptors and subsequently activates NF-κB. NF-κB regulates expression of a majority of inflammatory factors, such as IL-1β and IL-18 (Liu et al., 2016[11]). But, initially generated IL-1β and IL-18 are inactivated and rely on further processing by NLRP3/caspase-1 cascades for their maturation (Pan et al., 2016[12]). The present study adds novel evidence that Dex also suppresses NLRP3/caspase-1 cascades to inhibit inflammatory response induced by LPS/ATP.

There is evidence indicating that various biological effects of Dex is associated with its modulating transcription factor c-Fos. Previous study found that intrathecal administration of Dex reduces the formalin-induced paw-flinching behaviour in rats, which is due to the analgesic effects of Dex via a α2-receptor dependent manner as well as the inhibition of c-Fos activation (Zhang et al., 2013[24]). Inhibition of c-Fos activation by Dex alleviates postoperative cognitive dysfunction by abnormal neuron excitation in aged rats as well (Xiong et al., 2016[22]). The present study found that c-Fos nuclear protein levels in HMC3 cells were notably increased in HMC3 cells under the stimulation of LPS/ATP, but treatment with Dex abolished the increase in c-Fos nuclear protein levels, as indicated by both Western blot and IF assays. Luciferase report assay further revealed that c-Fos can bind to the promoter region of *NLRP3* gene and positively regulate its expression. Thus, it was suggested that Dex inhibiting c-Fos nuclear protein levels promoted by LPS/ATP blocks the up-regulation of NLRP3. This suggestion is supported by our Co-IP and PCR studies, in which Dex decreased the amount of c-Fos that binds to NLRP3 under the stimulation of LPS/ATP. 

The present study revealed that Dex inhibits inflammation in microglia cells under stimulation of LPS and ATP by c-Fos/ NLRP3/caspase-1 cascades, which adds new understanding of the anti-inflammatory mechanism of Dex. The outcome thus revealed the potential neuroprotective effect of Dex against AD. 

## Notes

Hu Li and Xueping Zhang contributed equally as first authors.

Jianyan Chen and Tao Gao (Department of Anesthesiology, Shenzhen Shajin Hospital Affiliated to Guangzhou Medical University, Shenzhen 518100, China; E-mail: gaotao197771@163.com) contributed equally as corresponding authors.

## Conflict of interest statement

The authors declare no conflicts of interest.

## Acknowledgements

The authors thank Dr Chu-hong Xu (Department of Clinical Pharmacology, Union Hospital, Huazhong University of Science and Technology, Wuhan, China) for his assistance with analysis of the data.

## Funding

This study was supported by the Shenzhen Municipal Commission of science and technology innovation (Grant No. JCYJ20150402152005631).

## Supplementary material

To this publication supplementary material is added .

## Supplementary Material

Supplementary material
