# Supplementary material for: Dexmedetomidine inhibits inflammation in microglia cells under stimulation of LPS and ATP by c-Fos/NLRP3/caspase-1 cascades
Source: EXCLI J. 2018 Mar 22;17:302–11. doi: 10.17179/excli2017-1018 (PMC5938529; doi:10.17179/excli2017-1018)
Supplement: Supplementary material [file EXCLI-17-302-s-001.pdf]

EXCLI Journal 2018;17:302-311 – ISSN 1611-2156
Received: December 11, 2017, accepted: January 23, 2018, published: March 22, 2018

Supplementary material to

Original article:

DEXMEDETOMIDINE INHIBITS INFLAMMATION IN MICROGLIA CELLS UNDER STIMULATION OF LPS AND ATP BY C-FOS/NLRP3/CASPASE-1 CASCADES

Hu Li[1†], Xueping Zhang[2†], Mingfu Chen[3], Jianyan Chen[4#], Tao Gao[3#], Shanglong Yao[5]

[1] Department of Anesthesiology, Shenzhen Baoan Hospital Affiliated to Southern Medical University, Shenzhen 518100, China
[2] Department of Anesthesiology, Shenzhen People's Hospital, The Second Clinical Medical College, Jinan University, Shenzhen Anesthesiology Engineering Center, Shenzhen 518020, China
[3] Department of Anesthesiology, Shenzhen Shajin Hospital Affiliated to Guangzhou Medical University, Shenzhen 518100, China
[4] Department of Anesthesiology, Second People's Hospital of Futian District, Shenzhen 518029, China
[5] Department of Anesthesiology, Union Hospital, Tongji Medical College, Huazhong University of Science and Technology, Wuhan 430022, China

[†] Both authors contributed equally to this work.
[#] Corresponding authors, E-mail: chenjianyan@yahoo.com (J. Chen), gaotao197771@163.com (T. Gao)

http://dx.doi.org/10.17179/excli2017-1018

This is an Open Access article distributed under the terms of the Creative Commons Attribution License (http://creativecommons.org/licenses/by/4.0/).

EXCLI Journal 2018;17:302-311 – ISSN 1611-2156
Received: December 11, 2017, accepted: January 23, 2018, published: March 22, 2018

Figure 1A: Cell viability tests

Groups	Test1*	Test2	Test3	Average	Compared to control
**A**	0.466	0.423	0.522	0.470333333	1
**B**	0.362	0.274	0.307	0.314333333	0.651266766
**C1**	0.309	0.281	0.295	0.295	0.627214741
**C2**	0.367	0.333	0.355	0.351667	0.747696669
**C3**	0.394	0.403	0.419	0.405333	0.861800142

*The tested value is absorbance. Test (1-3) indicates that one experiment was repeated for 3 times.
Note: The absorbance in the table is equal to original test values deducting the average background value (0.023). For example in the group A of Test1, 0.489-0.023=0.466

Figure 1B: Extracellular concentrations of inflammatory factors

IL-1β concentration

Groups	Test1*	Test2	Test3	Average	Concentrations (pg/ml)
**A**	0.735	0.648	0.607	0.663333	101.7796667
**B**	2.012	2.676	2.935	2.541	423.49024
**C1**	2.096	2.327	2.658	2.360333333	392.4806133
**C2**	1.867	1.543	1.526	1.645333333	269.7580133
**C3**	1.314	1.029	1.005	1.116	178.90324

*The tested value is absorbance.
Note: a, b, c, d, e, f, g and h indicate the test values of the standard substance of IL-1β at 500, 250, 125, 62.5, 31.25, 15.625, 7.8, and 0 pg/ml, respectively.
The absorbance in the table is equal to original test values deducting the average background value (0.028). For example in the group A of Test1, 0.763-0.028=0.735

IL-18 concentration

Groups	Test1*	Test2	Test3	Average	Concentrations (pg/ml)
**A**	0.463	0.408	0.577	0.482666667	147.4943467
**B**	0.846	0.748	0.971	0.855	281.5269
**C1**	0.893	0.764	0.781	0.812666667	266.2877467
**C2**	0.767	0.645	0.708	0.706666667	228.1298667
**C3**	0.502	0.536	0.592	0.543333333	169.3331333

*The tested value is absorbance.
Note: a, b, c, d, e, f, g and h indicate the test values of the standard substance of IL-18 at 1000, 500, 250, 125, 62.5, 31.25, 15.625, and 0 pg/ml, respectively.
The absorbance in the table is equal to original test values deducting the average background value (0.028). For example in the group A of Test1, 0.491-0.028=0.463

Figure 1C: Relative protein levels

NLRP3 protein level

Groups	Test1*	Test2	Test3	Average
**A**	0.036	0.079	0.022	0.045667
**B**	0.206	0.269	0.324	0.266333
**C1**	0.178	0.153	0.177	0.169333
**C2**	0.049	0.083	0.103	0.078333
**C3**	0.044	0.071	0.095	0.07

*The value is the ratio of optical density of NLRP3 to that of GAPDH.

Noncleaved caspase-1

Groups	Test1*	Test2	Test3	Average
**A**	0.275	0.179	0.237	0.230333
**B**	0.07	0.01	0.03	0.036667
**C1**	0.09	0.05	0.02	0.053333
**C2**	0.08	0.03	0.04	0.05
**C3**	0.31	0.28	0.21	0.266667

*The value is the ratio of optical density of noncleaved caspase-1 to that of GAPDH.

Cleaved caspase-1

Groups	Test1*	Test2	Test3	Average
**A**	0.09	0.06	0.03	0.06
**B**	0.32	0.24	0.29	0.283333
**C1**	0.175	0.195	0.226	0.198667
**C2**	0.082	0.176	0.123	0.127
**C3**	0.097	0.074	0.076	0.082333

*The value is the ratio of optical density of cleaved caspase-1 to that of GAPDH.

EXCLI Journal 2018;17:302-311 – ISSN 1611-2156
Received: December 11, 2017, accepted: January 23, 2018, published: March 22, 2018

Figure 2A: Relative protein expression

Groups	Test1*	Test2	Test3	Average
**A1**	0.155	0.136	0.093	0.128
**A2**	0.133	0.104	0.092	0.109667
**A3**	0.081	0.056	0.072	0.069667
**A4**	0.043	0.024	0.031	0.032667

*The value is the ratio of optical density of c-Fos to that of histone.

Figure 2B: Relative luciferase activity

Groups	Test1*	Test2	Test3	Average
**A**	0	0.12	0.25	0.123333
**B**	11.75	13.52	16.71	13.99333
**C**	2.61	3.86	1.43	2.633333
**D**	7.83	6.32	6.54	6.896667
**E**	13.21	11.32	10.57	11.7

*The value is relative luciferase activity.

EXCLI Journal 2018;17:302-311 – ISSN 1611-2156
Received: December 11, 2017, accepted: January 23, 2018, published: March 22, 2018

Figure 3A: c-Fos protein levels in cell nucleus

Groups	Test1*	Test2	Test3	Average
**A**	0.146	0.109	0.137	0.130667
**B**	0.045	0.037	0.021	0.034333
**C**	0.246	0.225	0.199	0.223333
**D**	0.016	0.006	0.012	0.011333
**E**	0.088	0.033	0.069	0.063333

*The value is the ratio of optical density of c-Fos to that of histone.

Figure 3A: Relative protein levels of NLRP3

Groups	Test1*	Test2	Test3	Average
**A**	0.046	0.078	0.084	0.069333
**B**	0.038	0.023	0.027	0.029333
**C**	0.214	0.284	0.271	0.256333
**D**	0.046	0.013	0.036	0.031667
**E**	0.19	0.11	0.14	0.146667

*The value is the ratio of optical density of NLRP3 to that of GAPDH.

Figure 3B: Fluorescence intensity of c-Fos

Groups	Test1*	Test2	Test3	Average
**A**	4.02	3.74	3.11	3.623333
**B**	2.17	2.53	1.94	2.213333
**C**	7.47	4.88	5.99	6.113333
**D**	1.76	1.22	2.17	1.716667
**E**	2.22	1.34	1.52	1.693333

*The value is the fluorescence intensity.

Figure 3C: Relative mRNA levels of *NLRP3*

Groups	Test1*	Test2	Test3	Average
**A**	1.192	0.916	1.081	1.063
**B**	0.658	0.595	0.358	0.537
**C**	2.57	2.91	1.89	2.456667
**D**	0.247	0.596	0.149	0.330667
**E**	1.57	0.941	0.879	1.13

*The value is relative mRNA expression level of *NLRP3.*

EXCLI Journal 2018;17:302-311 – ISSN 1611-2156
Received: December 11, 2017, accepted: January 23, 2018, published: March 22, 2018

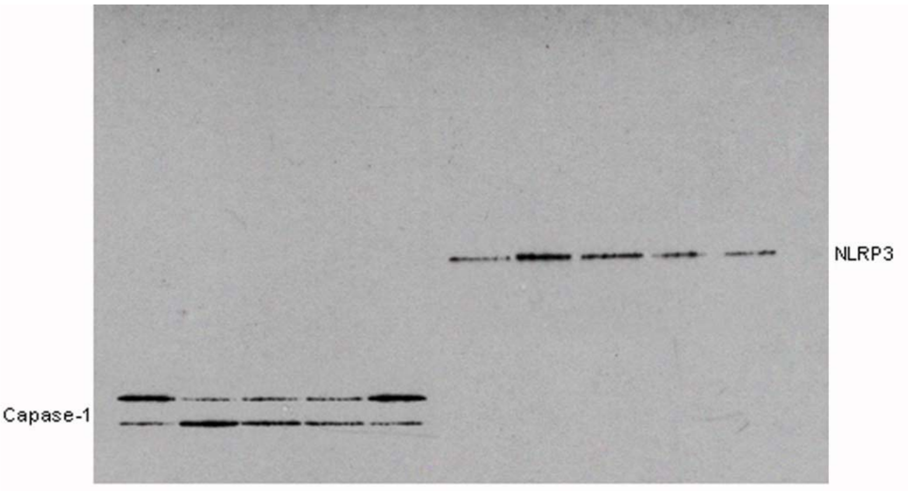

Fjgure-1C

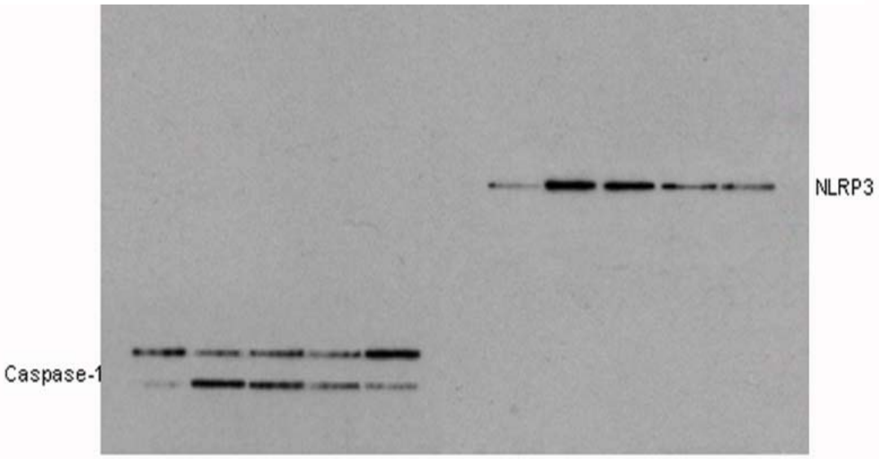

Figur e 1C

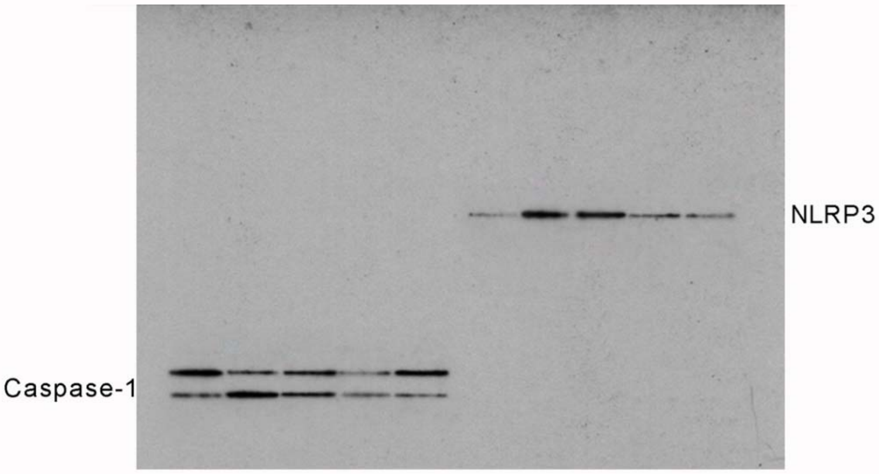

Figure-1C

EXCLI Journal 2018;17:302-311 – ISSN 1611-2156
Received: December 11, 2017, accepted: January 23, 2018, published: March 22, 2018

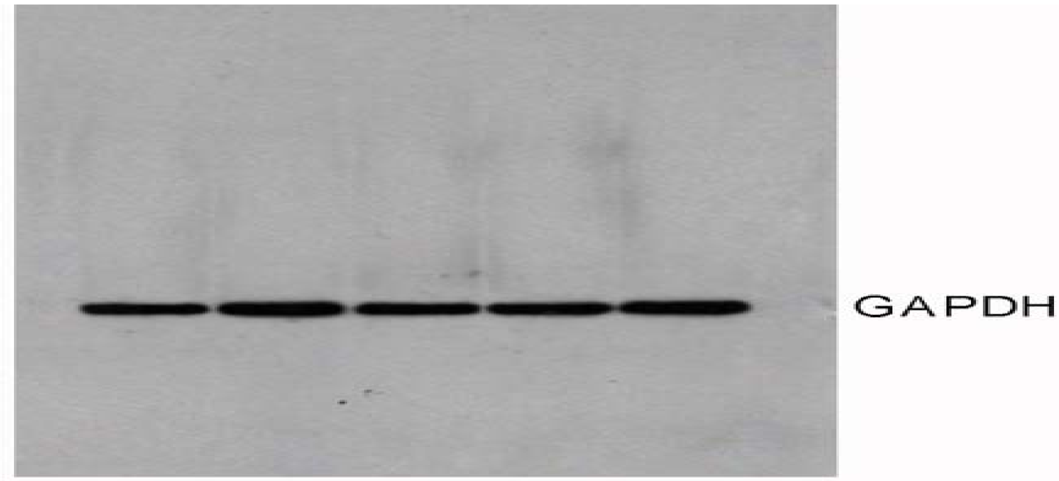

Figure-1C

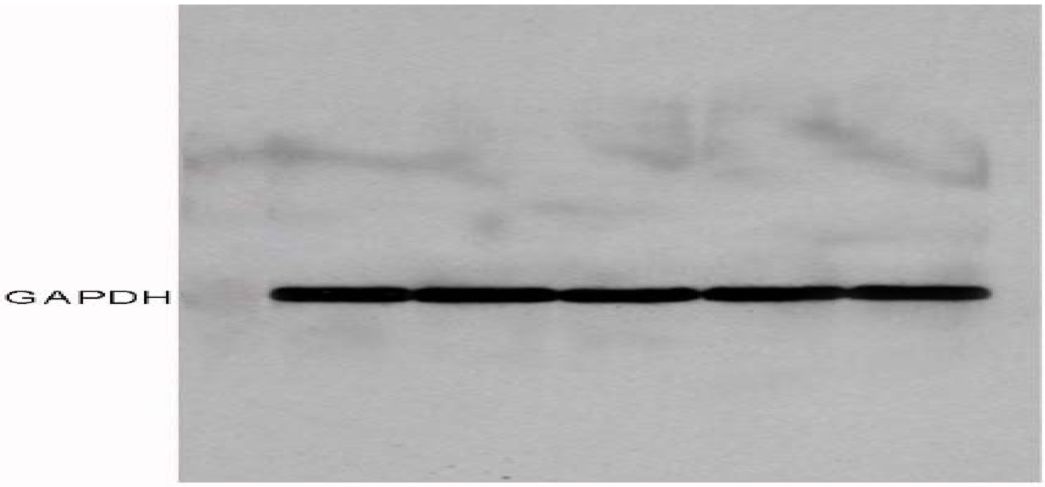

Figure-1C

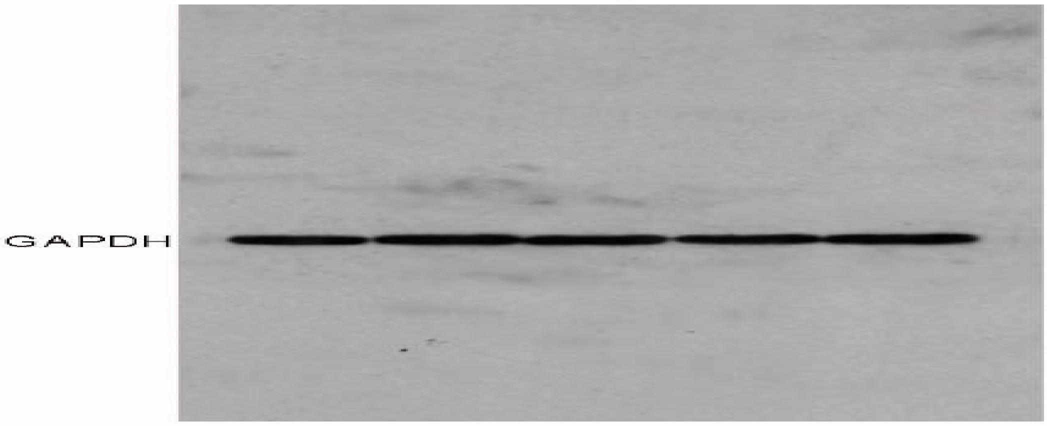

Figure-1C

EXCLI Journal 2018;17:302-311 – ISSN 1611-2156
Received: December 11, 2017, accepted: January 23, 2018, published: March 22, 2018

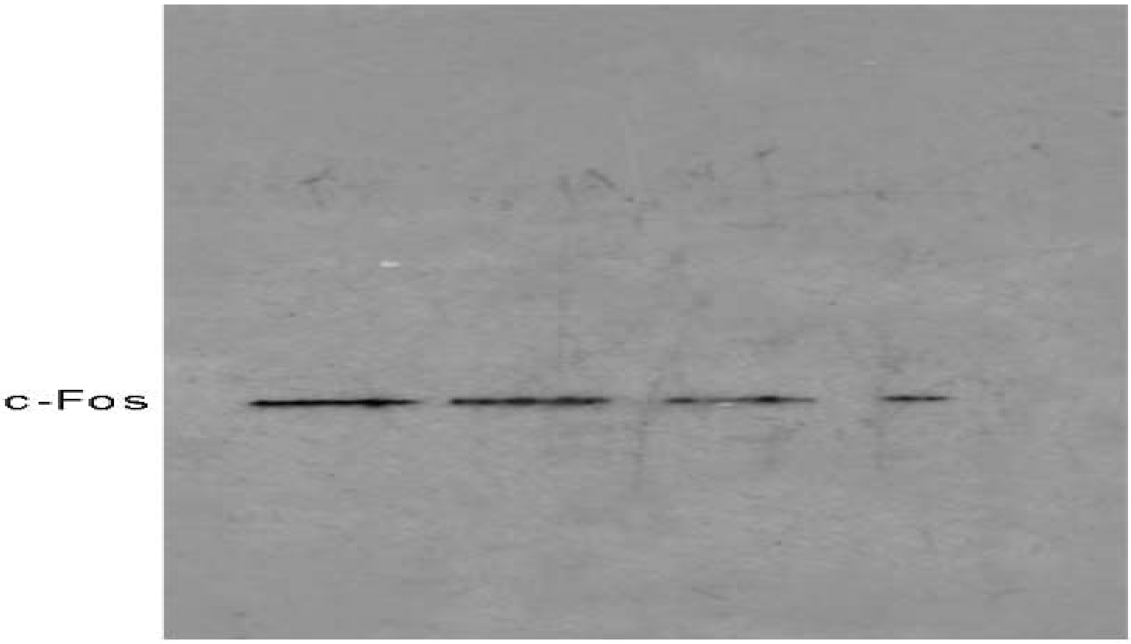

Figure-2A

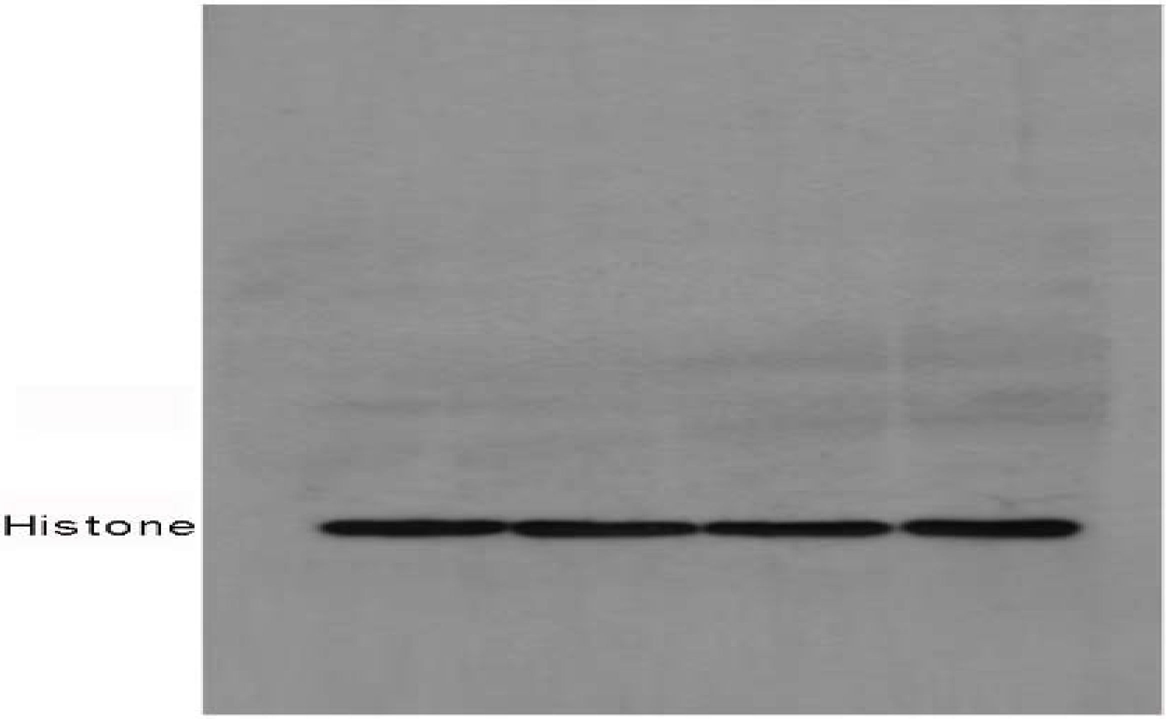

Figure-2A

EXCLI Journal 2018;17:302-311 – ISSN 1611-2156
Received: December 11, 2017, accepted: January 23, 2018, published: March 22, 2018

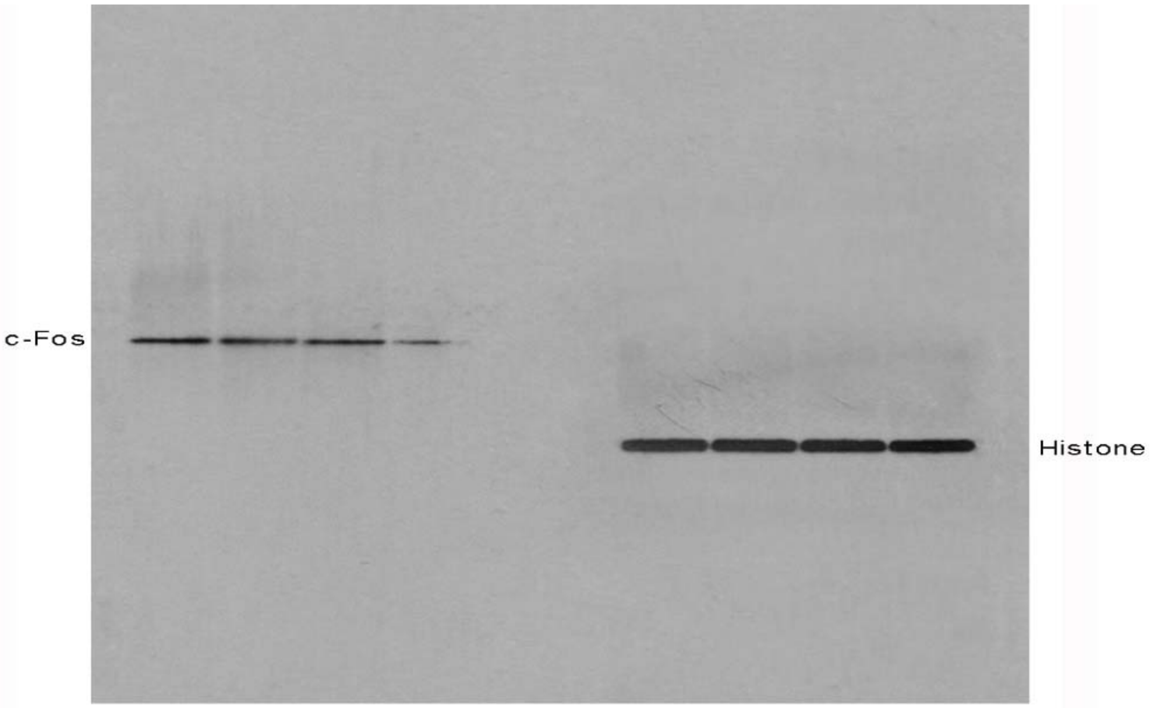

Figure-2A

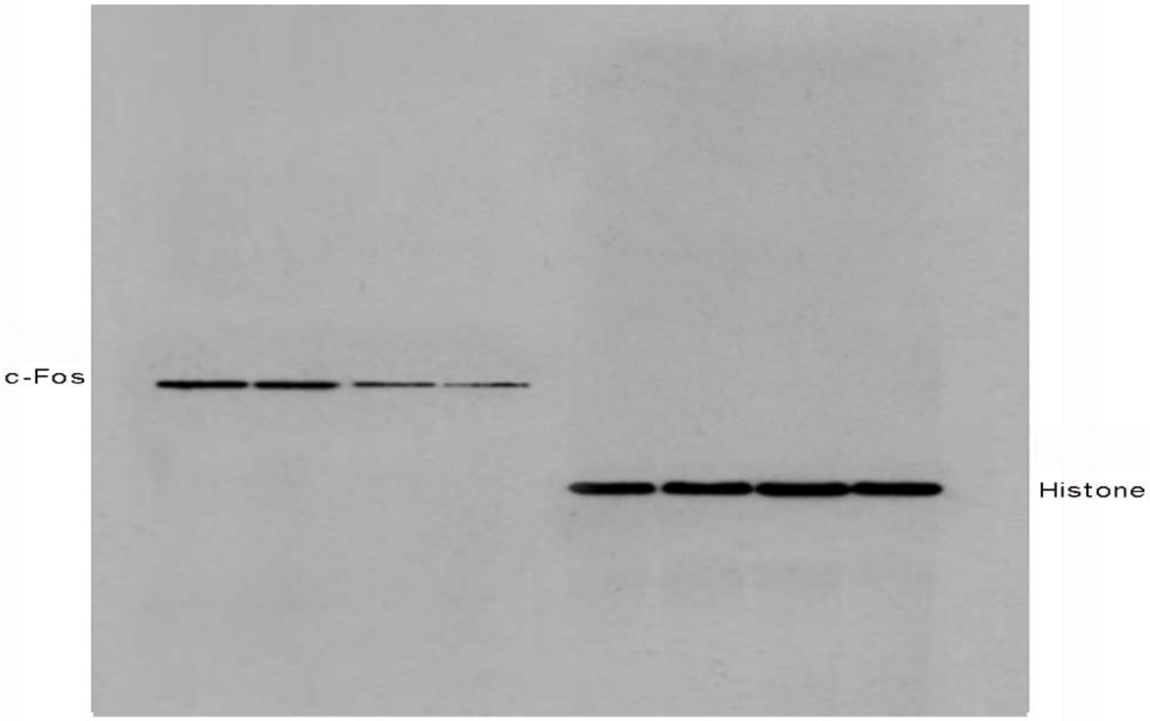

Figure-2A

EXCLI Journal 2018;17:302-311 – ISSN 1611-2156
Received: December 11, 2017, accepted: January 23, 2018, published: March 22, 2018

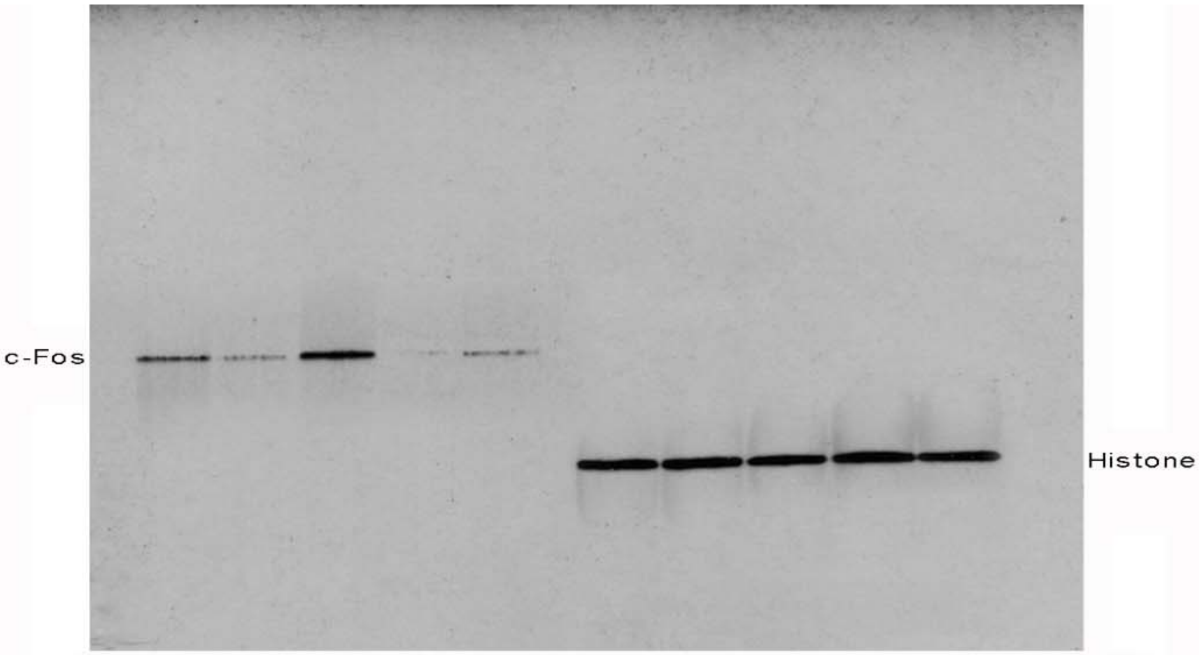

Figure-3A

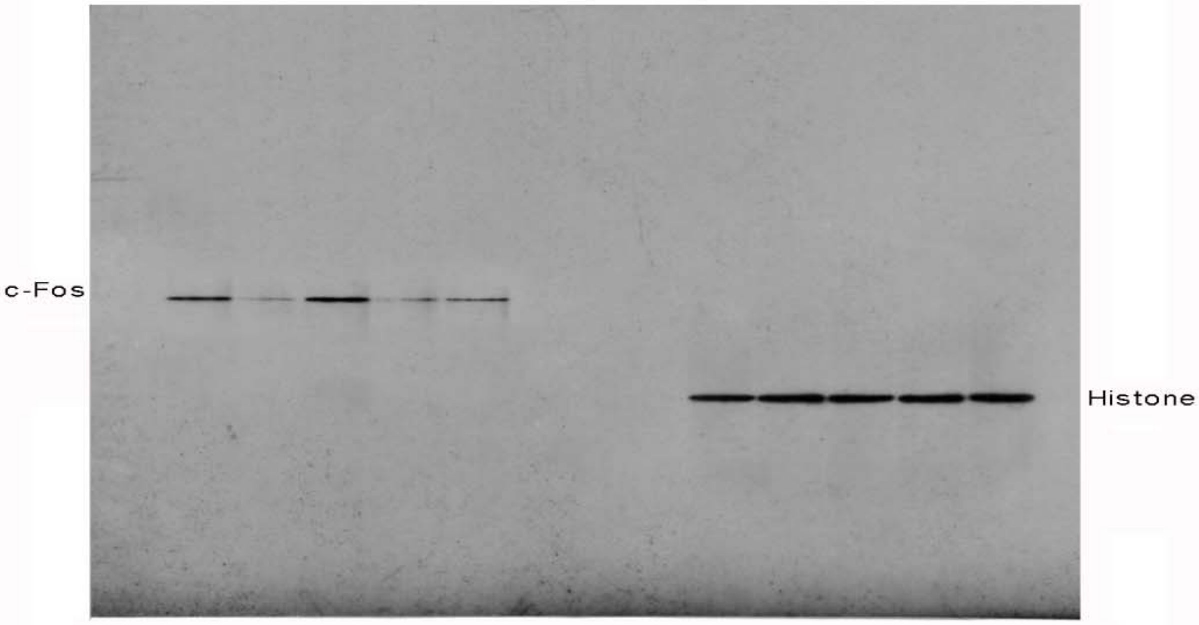

Figure-3A

EXCLI Journal 2018;17:302-311 – ISSN 1611-2156
Received: December 11, 2017, accepted: January 23, 2018, published: March 22, 2018

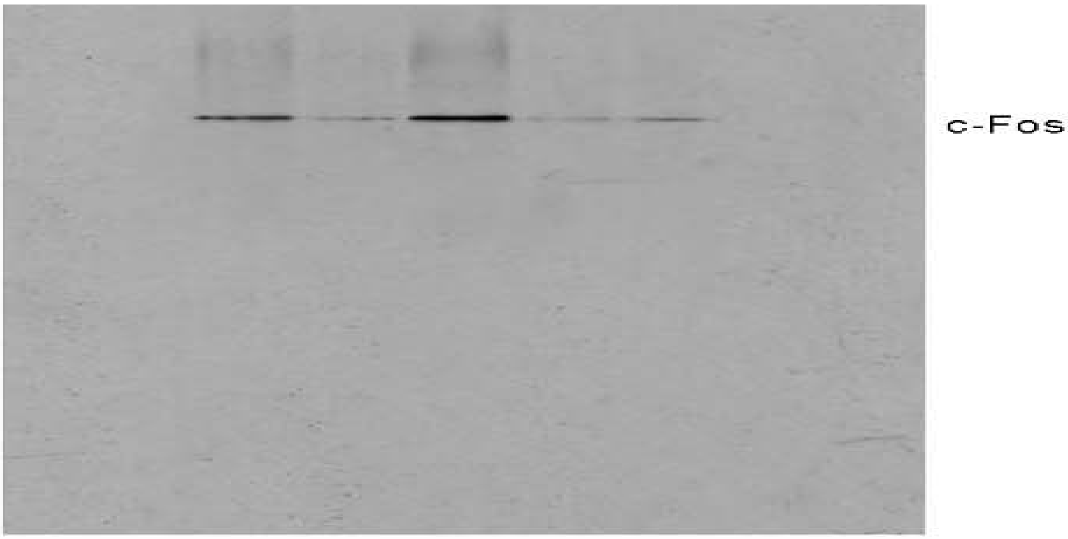

Figure-3A

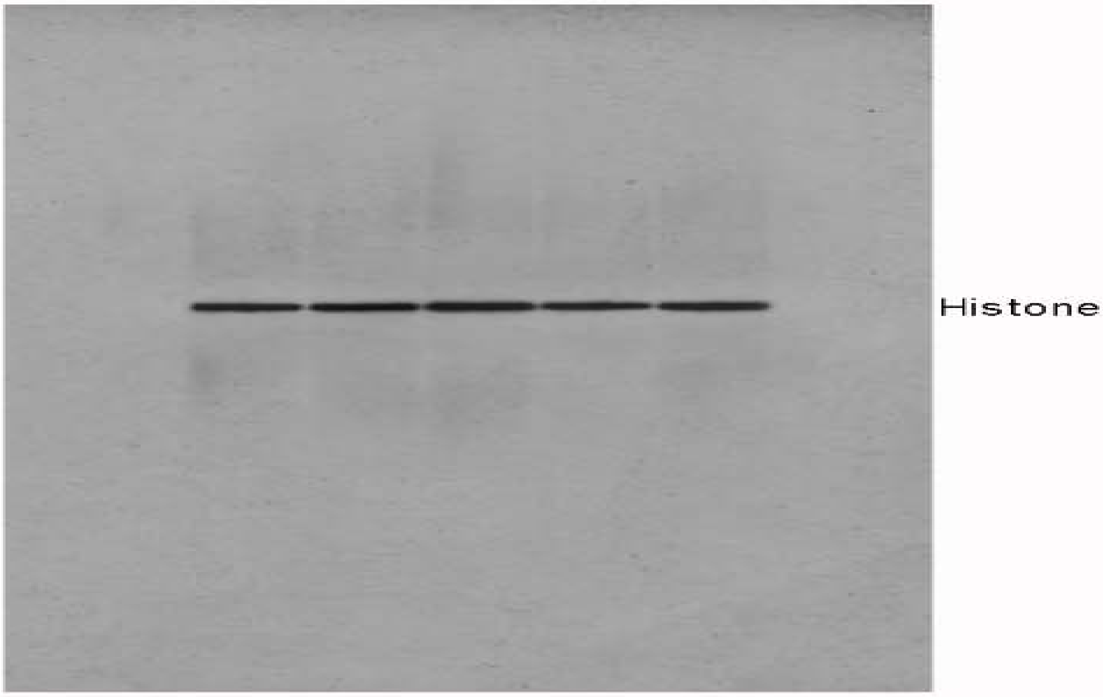

Figure-3A

EXCLI Journal 2018;17:302-311 – ISSN 1611-2156
Received: December 11, 2017, accepted: January 23, 2018, published: March 22, 2018

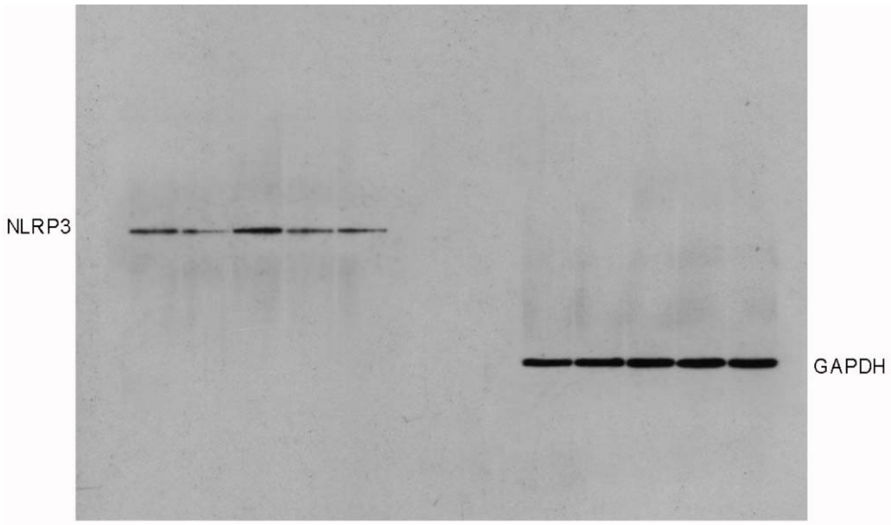

Figure-3A

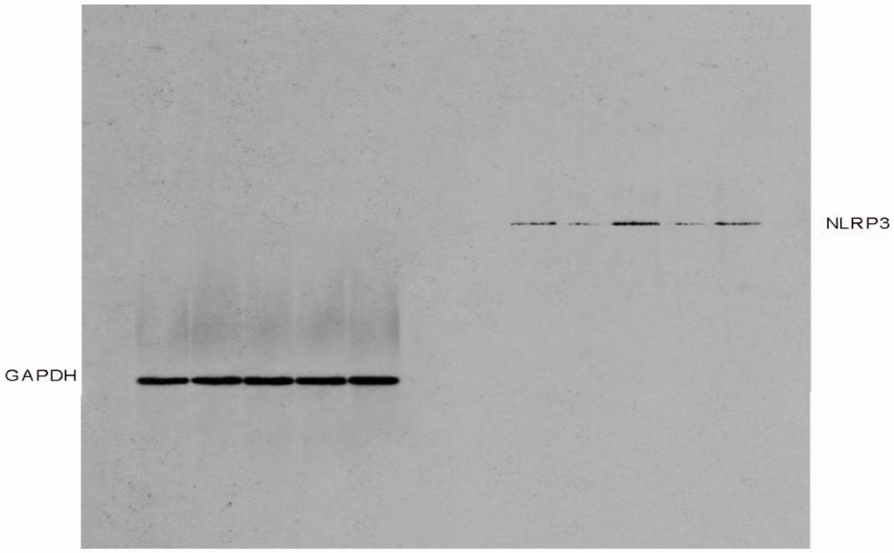

Figure-3A

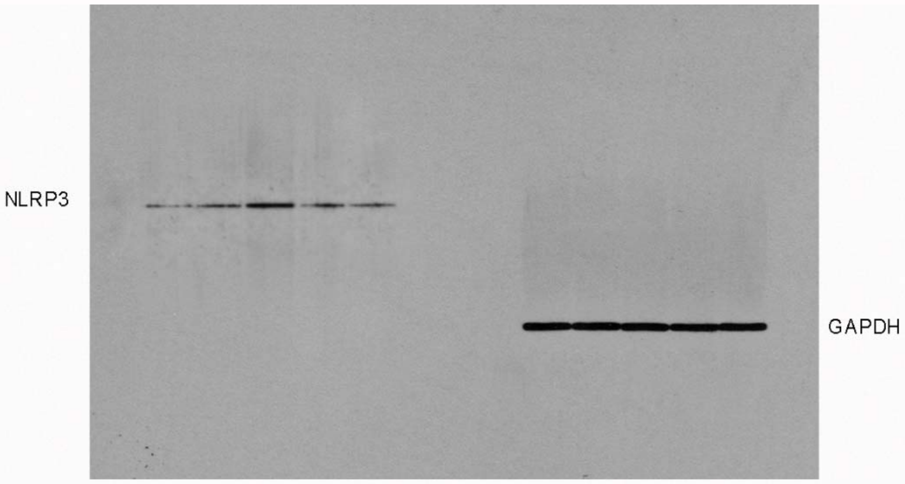

Figure 3A
